# Expression of BMP4 in myocardium and vascular tissue of obese mice

**DOI:** 10.1186/s12950-015-0047-6

**Published:** 2015-02-07

**Authors:** Ting Wu, Qiu-Yang Ling, Cheng Zhong, Tian-Xiao Wang, Lu-Lu Wang, Xiao-Ying Wang, Zhao-Liang Su, Gang-Jun Zong

**Affiliations:** Department of Cardiology, Wuxi Clinical Hospital, Anhui Medical University, Wuxi, Jiangsu Province China; Department of Cardiology, 101 Hospital of PLA, Wuxi, Jiangsu Province China; Central Laboratory, Zhenjiang No.4 People’s Hospital, Zhenjiang, Jiangsu Province China

**Keywords:** BMP4, Obesity, Inflammation, Cardiovascular disease

## Abstract

**Background:**

Obesity is regarded as a risk factor for cardiovascular disease. Bone morphogenetic protein 4 (BMP4) is a proinflammatory and profibrotic factor, and the reduced expression of this molecule in obese mice seems to be inconsistent with the known proinflammatory effects of obesity. Therefore, we studied BMP4 expression and inflammation in the myocardial tissue and aortas of obese mice.

**Methods and Results:**

Four-week-old *ob/ob* mice were used as the experimental group, and C57BL/6 mice comprised the control group. Animals were sacrificed after a 12-week full diet, and then the blood, heart, abdominal aorta, and inguinal adipose tissue were collected. The expression of BMP4 mRNA and protein in the heart and aorta was significantly higher in the experimental group than in the control group, but expression was lower in adipose tissue. Inflammation measured by the expression of IL-1β and IL-9 mRNA and protein and Smad1 and phosphorylated Smad1/5/8 protein in the heart and aorta was higher in the experimental group than in the control group. In addition, the expression of BMP4 in the serum was significantly higher in the experimental group than in the control group.

**Conclusion:**

BMP4 is significantly overexpressed in the myocardial tissue and aortas of obese mice, and mediates local inflammatory responses.

## Introduction

Observational epidemiological studies have documented that obesity is associated with cardiovascular risk factors, and is becoming one of the most important determinants of health and the quality of life in the global burden of disease [1]. Research has shown that obesity is a pathological state that can lead to local and systemic chronic oxidative stress and inflammation, and prolonged periods of obesity can result in artery endothelial dysfunction culminating in cardiovascular disease [2,3]. Excess adipose tissue is a characteristic manifestation of obesity; however, the relationship between excess adipose tissue and cardiovascular disease is still unknown.

Recently, a number of studies have shown that bone morphogenetic protein 4 (BMP4) mediates adipocyte differentiation of pluripotent stem cells, and is closely related to body adipose tissue volume. The expression levels of BMP4 are higher in the adipose tissue of individuals with a lower body mass index than those with a high body mass index [4-6]. BMP4 impairs endothelial function via the activation of vascular nicotinamide adenine dinucleotide phosphate oxidase, which enhances the production of peroxides and reduces endothelial bioavailability of nitric oxide [7-9]. These findings indicate that BMP4 is a proinflammatory factor, although its reduced expression in adipose tissue seems to be inconsistent with the known proinflammatory effects of obesity. We proposed that the excess adipose tissue associated with obesity might lead to negative feedback regulation of the BMP4-mediated adipocyte differentiation of pluripotent stem cells. Subsequently, surplus BMP4 released into the blood could affect the vascular endothelium and the myocardium, leading to cardiovascular disease. In the present study, we tested our hypothesis by measuring the expression levels of BMP4 in the myocardial tissue and aortas of obese mice. These data may assist in the identification of effective therapeutic targets for clinical prevention of obesity-associated cardiovascular disease.

## Materials and methods

### Materials

Four-week-old healthy male *ob/ob* and C57BL/6 mice (18–24 g) raised in specific pathogen-free conditions were purchased from Nanjing Qingzilan Technology (Nanjing, China). This study was approved by Animal Care and Use Committee of the Anhui Medical University and all animals received care in compliance with the Guide for the Care and Use of Laboratory Animals published in 1988 by The National Academies. Rabbit BMP4, IL-1β, and IL-9 polyclonal antibodies were purchased from Abcam (Cambridge, England). Rabbit Smad1 and phosphorylated Smad1/5/8 (p-Smad1/5/8) polyclonal antibodies were purchased from Cell Signaling Technology (Shanghai, China). Polymerase chain reaction (PCR) and real-time quantitative PCR kits were purchased from Takara Bio (Otsu, Japan). Enzyme-linked immunosorbent assay (ELISA) kits were purchased from Abcam (Cambridge, England).

### Methods

#### Animal models and groups

*Ob/ob* mice and C57BL/6 mice were chosen as the experimental group and control group, respectively, with eight animals in each group. Mice were fed in rooms maintained at 20–25°C, with 40–50% relative humidity. After 12 weeks of routine feeding and freely available water, blood was collected by heart puncture and the mice were sacrificed after they were weighed. Subsequently, the heart, abdominal aorta, and inguinal adipose tissue were collected.

#### Sample preparation

The cardiac apex (5 mm), the proximal part of the abdominal aorta (1 cm), and inguinal adipose tissue were embedded in a paraffin block and cut into 4 μm serial sections, so that each section contained all three tissues. Hematoxylin and eosin (HE) staining or immunohistochemical staining of BMP4 was performed on these sections. The remaining tissues were prepared for real-time fluorescence quantification and western blot detection of BMP4, IL-1β, IL-9 and Smad1.

#### Semi-quantitative detection of BMP4 protein by immunohistochemistry

Sections were dewaxed, rehydrated, and washed with phosphate-buffered saline (PBS) for 5 min, before immersion in 0.01 mol/L sodium citrate buffer (pH 6.0) and incubation in a water bath at 92–98°C for 15 min. Subsequently, the sections were left to cool naturally and were then washed with PBS for 5 min at room temperature. Sections were immersed in 3% H_2_O_2_ for 10 min and then washed with PBS. After the addition of serum diluted in PBS, sections were incubated at 37°C for 20 min. Anti-BMP4 antibody was diluted 1:200 in PBS containing serum, added to the sections, and incubated at 4°C overnight. The next day, the sections were washed five times in PBS before incubation with 1:200 diluted biotin-conjugated donkey anti-rabbit IgG at 37°C for 30 min, followed by treatment with 3,3′-diaminobenzidine, and counterstaining with hematoxylin. Sections were dehydrated, cleared in xylene, and mounted under a cover slip. BMP4 expression was observed and photographed under an optical microscope.

#### Real-time fluorescence quantification of BMP4, IL-1β, IL-9 and Smad1 mRNA

Total RNA was isolated from tissue specimens using TRIzol reagent (Life Technologies, Gaithersburg, MD, USA), according to the manufacturer’s instructions. Total mRNA was reverse transcribed to cDNA, and the mRNA expression levels of *BMP4*, *IL-1β*, *IL-9*, and *Smad1* were measured by real-time fluorescence quantification. Primers (Table 1) were designed using Primer 5 software, and primer sequences were synthesized by the Shanghai ShengGong Biological (Shanghai, China).

**Table 1:** **Details of primers used in this study**

**Gene**	**Forward primer (5′–3′)**	**PCR product size (base pairs)**
	**Reverse primer (5′–3′)**	
*BMP4*	F:CGCCAGCCGAGCCAACAC	169
	R:GGTCCACCTGCTCCCGAAAGA	
*IL-1β*	F:TGTGATGTTCCCATTAGAC	131
	R:AATACCACTTGTTGGCTTA	
*IL-9*	F:CCAGCTTCCAAGTGCCACTGC	125
	R:TGCATGGTGGTATTGGTCATCTG	
*Smad1*	F:AAACAGGGCGATGAAGAAGA	216
	R:CCACACACGGCAGTAAATGA	
β-actin	F:ACACCCGCCACCAGTTCGC	198
	R:TCTGGGCCTCGTCACCCACAT	

#### Western blotting of IL-1β, IL-9, Smad1, and p-Smad1/5/8 protein

Tissues were homogenized, and then cells were lysed with an appropriate amount of lysis buffer containing 2% (wt/vol) SDS and 60 mM Tris HCl (pH 6.8). Proteins were extracted by centrifugation at 13,000 rpm for 30 min at 4°C. Protein concentration was adjusted with the addition of sample buffer and then incubated at 95°C for 5 min. Protein lysates were separated by SDS-PAGE and transferred to polyvinylidene difluoride membranes. Primary antibodies were diluted in PBS/Tween containing 5% milk powder and added to the membranes for incubation at 4°C overnight. Membranes were incubated in the appropriate secondary antibody at room temperature for 2 h, and proteins were then detected by radiography.

#### BMP4 protein ELISA

Serum levels of BMP4 were determined using cytokine ELISA kits in accordance with the manufacturer’s instructions. All samples were assessed in duplicate. The results were expressed in pg/mL, and the detection limit for BMP4 was 8.23 pg/mL.

#### Statistical analysis

Data were analyzed using the SPSS13.0 software package, and measurements are expressed as the mean ± standard deviation $\left(\overline{\mathrm{X}}\pm \mathrm{s}\right)$. Analysis of variance was used to estimate the difference between groups, and *P* < 0.05 was considered statistically significant.

## Results

### Establishment of the obese mouse model

After 12 weeks of ordinary feeding, the body weight of C57BL/6 mice increased from 18.2 ± 0.97 g to 24.3 ± 0.73 g (approximately 1.3-fold), whereas the body weight of *ob/ob* mice increased from 20.4 ± 1.89 g to 36.9 ± 1.47 g (approximately 1.8-fold).

**Figure 1:**
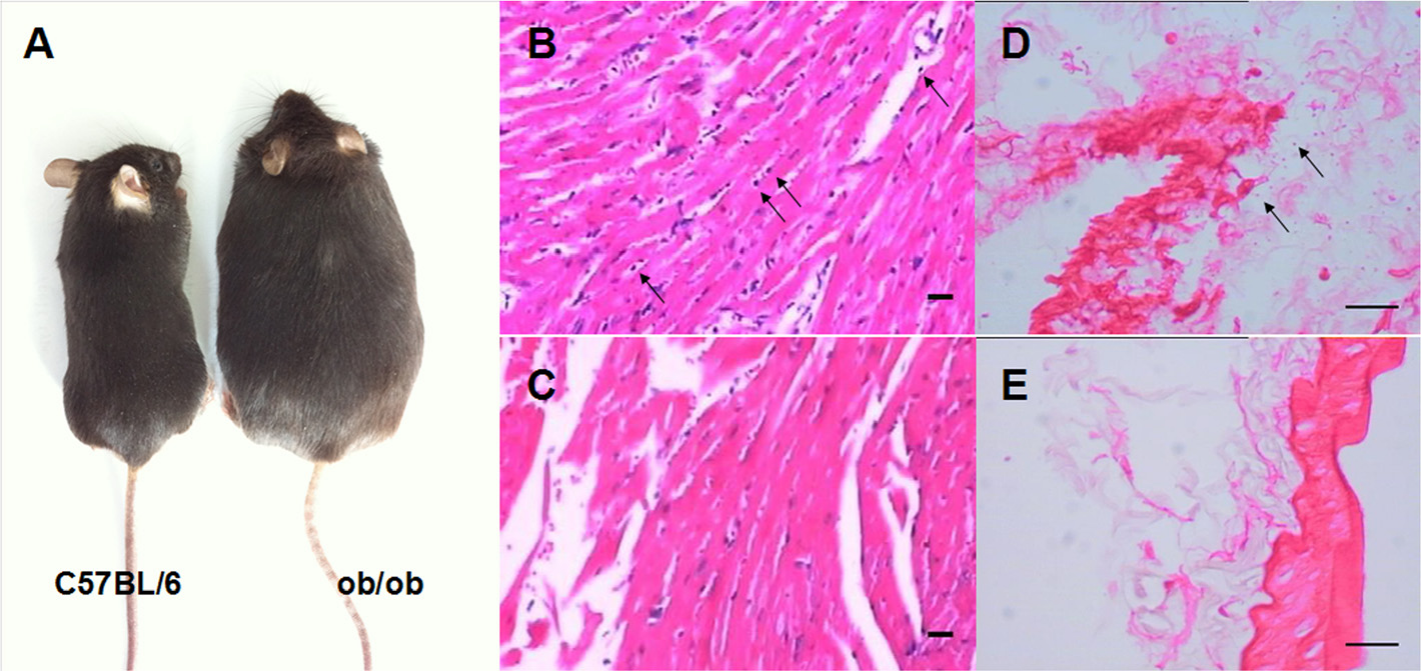
**General appearance and pathological findings in among two groups of animals (n = 8 for each group). A)** Comparison of general appearance of obesity mouse (*ob/ob*) and normal mouse (C57BL/6) at the study period. **B)** to **E)** The results of H&E stain showed low-grade inflammatory cell infiltration of the myocardium **(B, C)** and aorta **(D, E)** in the experimental group **(B, D)**. No inflammatory cells were observed in the control group **(C, E)**. (Scale bar: 20 μm.)

Successful obesity induction is defined as an increase in mouse body weight of more than 35% after feeding for 13 weeks [10]. After 12 weeks, 100% mice in our experimental group fit the criteria of obesity. Representative mice from the experimental group and the control group are shown in Figure 1A.

### HE staining of the myocardium and vasculature

HE staining revealed low-grade inflammatory cell infiltration of the myocardium and vasculature in the experimental group (Figure 1B,D). No inflammatory cells were observed in the control group (Figure 1C,E).

### Expression levels of BMP4 mRNA and protein

**Figure 2:**
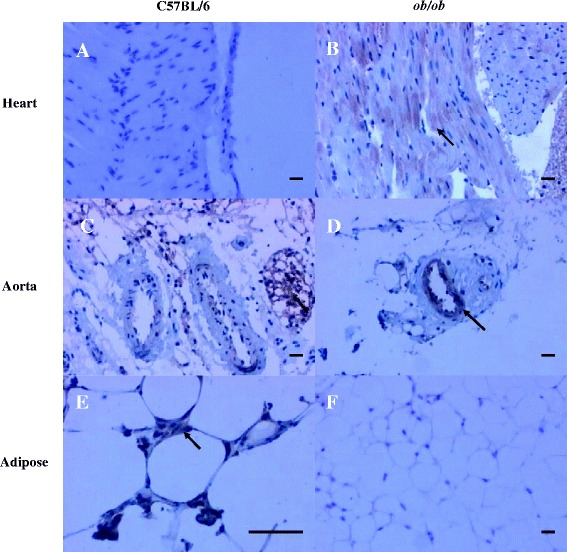
**Expression of BMP4 in heart, aorta and adipose tissue (n = 8 for each group). A)** to **D)**1 BMP4 expression in the heart **(A, B)** and **aorta (C, D)** was significantly higher in the experimental group **(B, D)** than in the control group **(A, C)**, especially in arterial endothelial cells **(D)**. **E)** and **F)** Showing the expression of BMP4 in adipose tissue was lower in the experimental group **(F)** than in the control group **(E)**. (Scale bar: 20 μm).

BMP4 expression in the heart and aorta was significantly higher in the experimental group than in the control group (Figure 2A-D). Cellular BMP4 expression was almost ubiquitous, particularly in arterial endothelial cells (Figure 2D). The expression level of BMP4 in adipose tissue was lower in the experimental group than in the control group (Figure 2C,E,F). Figure 2C and E (100× and 400× magnification, respectively) shows strong expression of BMP4 in the adipose tissue around the artery, denoted by the brown-colored stain.

**Figure 3:**
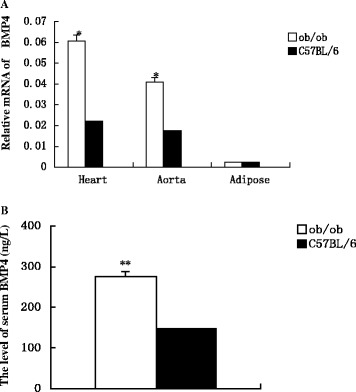
**The mRNA expressions of BMP4 in heart, aorta and adipose tissue, and the protein expression of BMP4 in serum (n = 8 for each group). A)** Showing the gene expression of BMP4 in heart and arteries was remarkably higher in experimental group than in control group (*P < 0.05), but there was no statistically significant difference in the *BMP4* mRNA expression levels in adipose tissue between the two groups (*P* > 0.05). **B)** The protein expression of BMP4 in the blood serum was significantly increased in the experimental group than in the control group (**P < 0.01).

*BMP4* mRNA expression levels in the heart and aorta were significantly higher in the experimental group than in the control group (*P* < 0.05). However, there was no statistically significant difference in *BMP4* mRNA expression levels in adipose tissue between the two groups (*P* > 0.05; Figure 3A).

The analysis of serum by ELISA revealed that BMP4 expression was significantly higher in the experimental group than in the control group (*P* < 0.05; Figure 3B).

### Expression of proinflammatory factors

**Figure 4:**
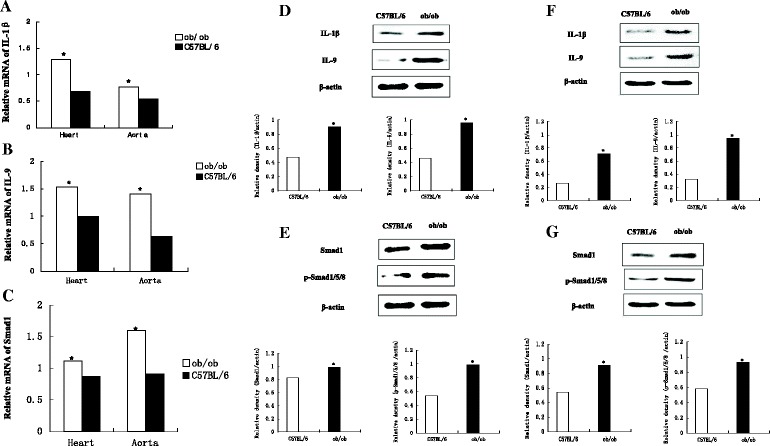
**The mRNA and protein expressions of inflammatory biomarkers and Smad1 in heart and aorta (n = 8 for each group). A)** to **C)** The mRNA expressions of IL-1β IL-9 and Smad1 were remarkably increased in the myocardium and aorta of the experimental group than the control group (*P < 0.05). **D)** to **G)** The protein expressions of IL-1β, IL-9, Smad1 and p-Smad 1/5/8 were significantly higher in the myocardium **(D, E)** and aorta **(F, G)** of animals in the experimental group than those in the control group (*P < 0.05).

To assess whether inflammation was present in the cardiovascular tissue of obese mice, we examined the expression of two proinflammatory factors: *IL-1β* and *IL-9*. Quantitative PCR demonstrated that *IL-1β* and *IL-9* were expressed at significantly higher levels in the myocardium and aorta of animals in the experimental group than those in the control group (*P* < 0.05; Figure 4A,B). Furthermore, western blotting demonstrated that IL-1β and IL-9 expression levels were also higher in the myocardium and aorta of animals in the experimental group than those in the control group (*P* < 0.05; Figure 4D,F).

The Smad pathway is known for BMPs [11]. Our data suggest that BMP4 overexpression in the heart and aorta of mice in the experimental group activated the Smad pathway, as the level of Smad1 in these mice was higher than in the control mice (P < 0.05, Figure 4C,E,G). In addition, the overexpression of BMP4 in the heart and aorta of mice in the experimental group increased the expression of p-Smad1/5/8 (P < 0.05, Figure 4E,G).

## Discussion

In the present study, we used *ob/ob* mice, which lack leptin and become obese under normal dietary conditions, enabling us to exclude the effects of a high-fat diet on the cardiovascular system. Majority of the fat in adult mice is contained in the belly, and is known as the white adipose tissue. Therefore, the acquisition of adipose tissue in mice is predominantly in the inguinal region. Our animal model revealed that BMP4, which mediates local inflammatory responses, was significantly overexpressed in the myocardial tissue and aortas of the obese mice.

We confirmed that BMP4 protein was expressed at lower levels in the adipose tissue of the obese mice than the control animals; however, there was no significant difference in mRNA expression between groups. Thus, we speculated that when the volume of adipose tissue is increased, BMP4 within the adipose tissue transfers into the blood, leading to decreased levels of BMP4 protein in the adipose tissue. In our experiments, BMP4 increased in expression in the serum of the obese mice, which was consistent with our hypothesis.

Following its release from adipose tissue into the blood, BMP4 causes inflammation of the myocardial tissue and aorta, thus contributing to cardiovascular disease. In this study, immunohistochemical analysis confirmed that the expression of BMP4 was significantly higher in the heart tissue of the obese mice than in the heart tissue of the normal mice. However, in the aortas of the obese mice, BMP4 had almost completely infiltrated into the endothelial cells. Such infiltration was scarcely detected in the blood vessels of the control mice. Expression levels of BMP4 mRNA were consistent with this conclusion. These findings suggest that an increased volume of adipose tissue mediates the release of BMP4, which indirectly promotes the inflammation of cardiovascular tissue.

Obesity promotes cardiovascular tissue inflammation, leading to a high incidence of cardiovascular disease in obese patients [12,13]. Many previous studies have examined the mechanisms of local and systemic inflammation in obese patients, but the influence of increased adipose tissue on the cardiovascular system has not been well studied. Our results demonstrated that as the volume of adipose tissue in the mice increased, so did BMP4 expression in the myocardial tissue and arteries. To verify the influence of increased BMP4 expression on cardiovascular tissue, we assessed the expression of the proinflammatory factors IL-1β and IL-9. The protein and mRNA levels of IL-1β and IL-9 were significantly increased in the myocardial tissue and aorta, which confirms the presence of inflammation in these tissues within obese mice.

Cardiovascular disease is essentially a chronic low-grade inflammatory disease, and the increased inflammatory response leads to a higher incidence of hypertension, atherosclerosis, and other cardiovascular diseases [14,15]. Smad1 is a signaling molecule downstream of BMP4. BMP4 promotes a local inflammatory response via Smad1/5/8 phosphorylation [16,17], and we detected increased levels of Smad1 and p-Smad1/5/8, which confirmed that local inflammation is mediated by BMP4. Taken together, our findings confirmed that BMP4 can mediate cardiovascular tissue inflammation, and is a bridge between increased adipose tissue and increased cardiovascular inflammation.

In the present study, we have demonstrated that BMP4 plays a key role in obesity-induced cardiovascular disease. Thus, BMP4 is expected to become a new target for the prevention and treatment of cardiovascular disease in obese patients. However, obesity and cardiovascular disease are both multifactorial diseases, and BMP4 likely represents just one of numerous pathological mechanisms. Therefore, further research is necessary to confirm its role.
